# The Current Role of Radiation in the Management of Cholangiocarcinoma—A Narrative Review

**DOI:** 10.3390/cancers16091776

**Published:** 2024-05-04

**Authors:** Saurav Verma, Natalie Grindrod, Daniel Breadner, Michael Lock

**Affiliations:** 1 Division of Medical Oncology, Department of Oncology, Schulich School of Medicine & Dentistry, Western University, London, ON N6A 3K7, Canada; saurav.verma@lhsc.on.ca (S.V.); natalie.grindrod@lhsc.on.ca (N.G.); daniel.breadner@lhsc.on.ca (D.B.); 2 London Regional Cancer Program, London Health Sciences Centre, London, ON N6A 5W9, Canada

**Keywords:** cholangiocarcinoma, chemotherapy, radiotherapy, systemic therapy, surgery, gallbladder, targeted treatment

## Abstract

**Simple Summary:**

Cholangiocarcinoma is a rare cancer with a dismal prognosis. The rate of recurrence after treatment of localized disease is high. In this review, we discuss the evidence regarding the use of radiation to improve outcomes in unresectable cholangiocarcinoma. We also discuss studies that have incorporated radiation before and after surgery. The review also briefly highlights recent developments in systemic therapy, especially targeted treatments and immunotherapy.

**Abstract:**

Cholangiocarcinoma (CCA) is a rare cancer of bile ducts. It is associated with a poor prognosis. The incidence of CCA is rising worldwide. Anatomical subgroups have been used to classify patients for treatment and prognosis. There is a growing understanding of clinically important distinctions based on underlying genetic differences that lead to different treatment options and outcomes. Its management is further complicated by a heterogeneous population and relative rarity, which limits the conduct of large trials to guide management. Surgery has been the primary method of therapy for localized disease; however, recurrence and death remain high with or without surgery. Therefore, there have been concerted efforts to investigate new treatment options, such as the use of neoadjuvant treatments to optimize surgical outcomes, targeted therapy, leveraging a new understanding of immunobiology and stereotactic radiation. In this narrative review, we address the evidence to improve suboptimal outcomes in unresectable CCA with radiation, as well as the role of radiation in neoadjuvant and postoperative treatment. We also briefly discuss the recent developments in systemic treatment with targeted therapies and immune checkpoint inhibitors.

## 1. Introduction

**Figure 1:**
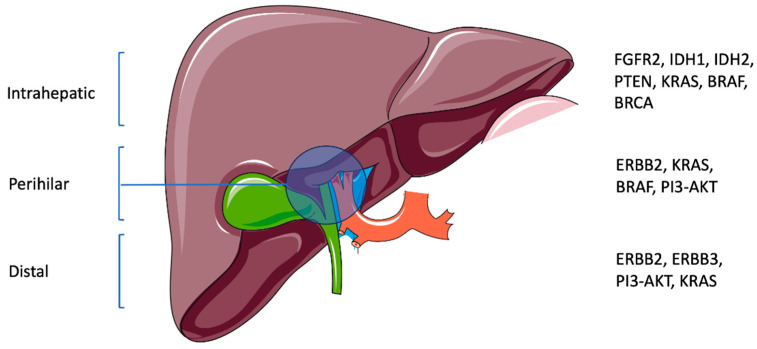
Anatomic subtypes of CCA with associated genetic aberrations (this schematic was created using Servier Medical Art templates, which are licensed under a Creative Commons Attribution 4.0 Unported License; https://smart.servier.com, accessed on 2 February 2024).

Cholangiocarcinoma (CCA) is a rare cancer that arises in the bile ducts. It is anatomically subdivided into intrahepatic CCA (iCCA) and extrahepatic CCA (eCCA), which includes perihilar CCA (pCCA) and distal CCA (dCCA). It is associated with poor outcomes, and its management is challenging. Recently, an increase in the incidence of CCA has been reported, predominantly in populations that do not possess the known risk factors [1]. Numerous reasons have been theorized for the observed increases, including more specific reporting resulting from the improved accuracy and availability of diagnostic tools [2]. The international classification of CCA varies between countries, which is a result of changing criteria within the International Classification of Disease for Oncology. Additionally, rising obesity rates and population migration between at-risk areas may be demographic-based reasons for the observed increase in incidence [2]. The 5-year overall survival (OS) is less than 10% and 0% for those with stage III and IV CCA, respectively. Of those who can undergo curative surgery, the 5-year OS is dismal, with rates between 15 and 40% [3]. The median OS (mOS) for unresectable CCA is 3–6 months [1]. Further, up to two-thirds of patients will have disease recurrence after surgery, most commonly in the liver, peritoneum, or abdominal lymph nodes [4]. As recently as 2017, surgery was understood to be the only possible curative approach, while chemotherapy and radiotherapies were used for palliative management to improve survival [1]. However, a greater understanding of the underlying genetics and novel treatments may offer therapeutic optimism (Figure 1). Recent studies have shown that there are other non-surgical treatments with associated improvements in survival.

In this narrative review, we discuss three major opportunities for intervention with radiation in the natural history of CCA patients: firstly, the management of unresectable or inoperable patients; secondly, neoadjuvant treatment; and lastly, postoperative treatment. This includes a review of practice-changing targeted therapies that have emerged based on an enhanced understanding of the genetic aberrations in various anatomic subtypes of CCA.

## 2. Unresectable or Inoperable CCA

Most patients with CCA present with unresectable/inoperable disease and have a bleak prognosis. Radiation has been examined as a potential treatment option when tumors cannot be surgically resected. In particular, early studies of radiation suggested that the non-invasive nature of this treatment modality may be of benefit. Radiation studies can be grouped by those using external beams with conventional doses, stereotactic body radiation therapy (SBRT), radionuclides, and proton therapy. A literature search (ClinicalTrials.gov headings: cholangiocarcinoma, bile duct cancer, inoperable, unresectable, radiation, radiotherapy; March 2024) revealed that only one phase III randomized controlled trial (RCT) has been completed, highlighting the difficulty in performing trials in this disease site and the lack of high-level data to guide management. Therefore, the available data come from 28 retrospective case series (Table 1) and epidemiological reviews. The Fudan University study is representative of the literature and is helpful in that it is a pure assessment of the benefits of radiation due to the fact that no other treatments were provided [5]. This study of 84 biopsy-proven unresectable iCCA patients found an overall response rate (ORR) of 37% with 8.6% complete remissions (CRs) and 28.5% partial responses (PRs). Compared to the ‘no EBRT’ group, the 1-year OS was 38.5% versus 16.4%, the 2-year OS was 9.6% versus 4.9%, and the mOS was 9.5 months versus 5.1 months. This study provided some of the first data that EBRT provides better outcomes. 

In addition to the retrospective data, there is support from large epidemiological research. The University of Pennsylvania study reviewed 3839 patients with iCCA in the Surveillance and Epidemiology and End Results (SEER) database [27]. This study found that radiation alone provided a significant improvement over no treatment (HR, 0.68; 95% CI, 0.59–0.77) with the best outcome found with radiation plus surgery (HR 0.40; 95% CI 0.34-0.47). This large landmark study encouraged the investigation of radiation for iCCA.

The only RCT in iCCA was conducted by the Federation Francophone de Cancerologie Digestive using a single-phase II randomized design (FFCD 99-02) investigating locally advanced CCA patients from multiple centers in France. Patients were randomized to chemoradiation (CRT) or chemotherapy (CT) (gemcitabine-oxaliplatin or gemcitabine-cisplatin (GC)). Radiation was 50 Gy in 25 daily fractions using external beam treatment. The study closed early in 2010 after a decade due to poor accrual. Though toxicity was lower in the CRT arm there was no signal of benefit in terms of progression free survival (PFS) or OS. The authors concluded that there was likely no additional benefit of radiation [6].

The failure of the French study to confirm the benefit seen in the SEER database may have been related to the technique and dose. Therefore, the assessment of SBRT is valuable, as it offers the potential for better identification of the disease both at consultation and during treatment, plus the ability to dose-escalate. A prospective trial comprising 10 CCA patients who underwent SBRT used a radiobiological dose selection protocol with a median dose of 36 Gy in six fractions [7]. The median survival was 15 months, which is double the survival rate (SR) that was found in the SEER database of similar patients [27], with a 1-year OS of 58%. Two of the ten patients had unexpected transient biliary obstruction, thought to be due to radiation edema. Other retrospective studies further examined the use of SBRT in advanced CCA and showed similar results [8,9,10]. Kozak et al. also examined SBRT in inoperable patients with a similar regimen and saw moderately improved results with a mOS of 23 months [11]. The mOS was 10 months for eCCA but 23 months for iCCA, possibly due to specific anatomic locations causing variable outcomes for SBRT. It is interesting that iCCA patients had such a higher mOS, especially compared to those studied by Zhang et al. (2022), who used SBRT (Cyber Knife) on iCCA patients, with similar outcomes to previous SBRT studies [15]. 

In a systematic review of 10 studies from 2019, the authors concluded that SBRT has potential as a therapeutic option for CCA [8]. Pooled OS was found to be 58.3% and 35.5% at 1 and 2 years, respectively. Notably, the 1-year local control (LC) was found to be 83.4% across studies. Although further studies are needed, the results are comparable to those observed with standard chemotherapy and are indicative of effective LC and acceptable treatment toxicity. Updating the literature review from this systematic review, their conclusions remain supported by recent studies [12,13]. 

There is one valuable study to note for dose escalation using SBRT with inoperable CCA that indicated that a biological equivalent dose (BED) greater than 80.5 Gy was a threshold ablative dose. This 79-patient case series demonstrated that the 3-year mOS increased from 38% to 73% when doses of more than 80.5 Gy were achieved. LC was similarly improved, rising from 45% to 78%. Importantly, this multicenter team was able to show that there were no significant radiation toxicities, providing support for the safety and efficacy of higher doses of radiation in iCCA [26]. In terms of dose escalation using techniques beyond external beam radiation include research using brachytherapy and proton therapy. A large retrospective review of the SEER database was carried out by Boothe et al., who mainly examined EBRT but also compared it to a subset of EBRT with brachytherapy [16]. They found that a brachytherapy boost increased mOS, but the use of this method has decreased over the last decade. This indicates that while EBRT alone improves survival, brachytherapy boost or dose escalation may provide additional benefit, but trials are lacking. 

Proton therapy may provide more focused treatment and higher doses. One cooperative group demonstrated the safety of proton beam therapy for iCCA [17]. Furthermore, their work has provided sufficient positive clinical outcomes to progress to prospective multicenter trials. One- and two-year OS was 69.7% and 46.5%, respectively, while one- and two-year PFS was 41.4% and 25.7%, respectively. However, at a median follow-up of 19 months, 53.9% of patients had distant metastases, with 12.8% having an isolated local failure and 2.6% having local and distant failures. There were similar results in another proton study with a moderately improved prognosis, possibly due to a higher radiation dose [18].

A meta-analysis of CCA patients who underwent either EBRT or selective internal radiation therapy (SIRT) (^90^Y-loaded glass microspheres) showed a meaningful OS and acceptable toxicities. The median survival time was 13.6 months and 12 months for EBRT and SIRT, respectively. When comparing treatments, it was found that current studies are too heterogeneous for there to be definitive conclusions [19]. Similar findings were found by Edeline et al., who completed a pooled analysis of various locoregional therapies, including EBRT and SIRT. The mean OS was 18.9 months and 14.1 months for EBRT and SIRT, respectively, which was slightly better than the previous analysis [20]. 

The variation in outcomes could be explained by the dose. Kumar et al. examined SIRT with a minimum dose of 190 Gy. Although the mOS was only 7 months, 25% of patients had excellent outcomes (mOS > 20 months) [21]. A study in 2018 retrospectively examined SIRT using a mean dose of 322 Gy. The results were remarkable, with a 100% response rate at three months. Furthermore, patients saw a median PFS (mPFS) of 12.7 months and a mOS of 28.6 months [23]. This finding is a substantial improvement compared to the mOS of 11.6 months observed in chemotherapy-alone patients. However, it must be noted that a high toxicity rate was detected in cirrhotic patients and Child–Pugh ≥A6 patients, indicating a need for further evaluation. SIRT added to chemotherapy may provide additional clinical improvement in CCA [22,24,25]. These encouraging results have prompted the initiation of a multicenter international RCT, SIRRCA (NCT02807181), that is investigating the value of adding Y90 resin microspheres prior to standard GC. This trial closed to recruitment in October 2022, and the results are pending.

These trials (Table 1) suggest that radiation improves local control, with both case series and epidemiological data demonstrating an OS advantage. However, the value of local treatment alone appears insufficient with both distant (predominantly) and local failures, as demonstrated by data from proton studies. These trials are heterogeneous, and the treatment regimens are varied. This limits an institution’s confidence in applying the data to individual patients with CCA of various anatomical locations and sizes. No guideline group has included radiation as a primary modality in iCCA. However, given similar level II data for other modalities and the lack of late liver or biliary toxicity, researchers are suggesting the inclusion of radiation in iCCA management to achieve the results seen in these case series. Despite the positive results seen in these multiple, primarily retrospective studies, there is a lack of any level I evidence and a lack of a standard dose regimen or target volume, and the small single-institution nature of the publications has dampened enthusiasm for both the use of radiation and its inclusion in guidelines. Therefore, accrual to high-level multicenter trials, using standard inclusion criteria and radiation dose regimens, is an important avenue to investigate in the management of iCCA.

## 3. Preoperative Radiation and Transplantation

The literature on the role of preoperative radiation and transplantation in CCA is controversial; it entails the greatest risks and yet may offer the best outcomes (Table 2). Given that a large proportion of patients are inoperable or borderline inoperable, the potential to get these patients into surgery is an active area of research. Consequently, additional treatments are being sought to improve the likelihood of successful surgeries and improve failure rates. 

The most optimistic data for neoadjuvant treatment is from the Mayo Clinic. Patients have been treated with planned, sequenced multimodality neoadjuvant treatment. The sequence consists of the following: a detailed patient selection process; EBRT with 5-FU for 2 weeks; 1 week after, brachytherapy with I-192; capecitabine; abdominal exploration for staging; if clear, liver transplantation for the patient. Patients are selected based on the likelihood of responding to therapy and survivability post-transplantation. The 5-year OS was 82% versus 21% for resection alone; the recurrence rate with neoadjuvant treatment was 12% versus 58% with resection alone, and 42% of explanted livers had no residual disease [28]. Updated Mayo Clinic data provide the same protocol, with the possibility of SBRT or PBT if brachytherapy is not possible. The Mayo protocol continues to show incredible results, with survival rates for intent-to-treat patients at 1, 5, and 10 years of 92%, 68%, and 60%, respectively. Looking at de novo and primary sclerosing cholangitis (PSC)-associated CCAs, the 1-, 5-, and 10-year survival rates (SR) were 91%, 58%, and 49% and 93%, 74%, and 67%, respectively [29]. 

The Mayo data are remarkable and have evidence of external validity. In 2012, Murad reviewed 12 US transplant centers [30]. The 5-year intention-to-treat (ITT) data indicated a mOS of 53% and a recurrence-free survival (RFS) of 67%. Similar to the Mayo data, 54% of explanted livers contained no residual disease. The authors noted that the results were adversely affected when patients not meeting eligibility criteria were included, especially those with tumor sizes greater than 3 cm. Based on these data and their own data, the Mayo Clinic recommends that patients with CCA arising in the setting of PSC receive neoadjuvant therapy and a transplant as the treatment of choice [28]. If it is not resectable, then neoadjuvant treatment should be considered with a view to transplantation, if possible, given the OS advantage in a subgroup of these patients.

Nonetheless, the Mayo Clinic protocol is a debated management technique that is not widely implemented. The reasons for this include that patients were highly preselected, the majority dropped out with progression, there was no direct comparison group, and there was a lack of clear management directions for borderline operable patients. For example, 20% of patients will stage positive despite a negative endoscopic ultrasound, most will develop cholangitis, and 20–40% will suffer a vascular complication. Furthermore, a trial conducted by a team in Toronto, Canada, failed to replicate the Mayo Clinic data [32]. Forty-three patients with lesions smaller than 3.5 cm were treated with CRT (EBRT and capecitabine). The patients were restaged and then received maintenance GC until transplantation. The results revealed that only 6 of the 43 patients received a transplant, with a 1-year SR of 83% and a 2-year SR of 55.6%. This was disappointing given that the Mayo Clinic reported a similar 1-year SR, but still higher at 91%, and then a very promising 5-year SR of 69% [28,37]. 

SBRT offers many benefits: concentrated doses to limited anatomical areas, short treatment durations, and minimized toxicities. These advantages spurred the University of Michigan trial to utilize SBRT, followed by neoadjuvant capecitabine for eCCA [33]. Due to the location of eCCA, surgery is challenging and often not a feasible option; fortunately, their findings suggest radiation as an efficacious alternative approach for those who have limited options. Of the 12 patients who underwent neoadjuvant therapy, 5 had at least a partial response (PR) with extensive tumor necrosis and fibrosis. Nine of the patients were eligible for transplant, but only six received a transplant, and 1 year after transplantation, 83% of patients had survived. In terms of long-term follow-up, the Universities of California and Wisconsin have performed neoadjuvant treatment for over three decades [35,36]. This retrospective review of 53 CCA patients (30 iCCA and 19 hCCA) treated between 1985 and 2019 assessed the value of a regimen of 40 Gy in five fractions given twice per week, followed by capecitabine, fluorouracil, or gemcitabine until transplant. Combined neoadjuvant CRT for hCCA and iCCA resulted in a 5-year OS of 88% and 100%. The 5-year OS was 9% and 41% for those who did not receive neoadjuvant CRT treatment. Chemotherapy alone or radiation alone did not result in a significant improvement in OS. This group concluded that multimodality neoadjuvant treatment is recommended, given the OS advantage. Furthermore, neoadjuvant treatment also appears to eliminate the prognostic factor of tumor size, which may allow more patients to access surgery. 

In conclusion, neoadjuvant radiation with chemotherapy followed by orthotropic liver transplantation, although not standard, is an acceptable approach in highly selected patients with early-stage CCA in the setting of PSC or small unresectable hilar CCAs.

### Preoperative Radiation and Resection

As previously stated, many patients cannot undergo surgery or are borderline, and those who undergo surgery often suffer a local recurrence or have close margins. Using neoadjuvant therapies could possibly downstage the disease and allow certain patients to be eligible for resection. In addition, using neoadjuvant therapies may aid in minimizing local recurrences and close margins.

A preoperative trial by MD Anderson Cancer Centre with nine eCCA cases (five pCCA and four dCCA) treated with 5-fluorouracil (5-FU) and EBRT found three cases with no residual disease and six with responses, and all had clear margins compared to 54% with surgery alone [31]. Importantly, there was no difference in the operative complication rate.

A more recent retrospective study using SIRT (Medical Internal Radiation Dose Model) in a neoadjuvant setting assessed patients with iCCA [34]. The mOS was 22 months, with 1-year OS at 60% and 2-year OS at 40%, and in comparison to other neoadjuvant treatments, it was not as beneficial. With the caveat that we are comparing across trials, when comparing unresectable cases in Table 1, there is similar OS, possibly better than neoadjuvant treatment. Even so, in inoperable cases, when CT was added, there was a significant benefit in OS and PFS. It could be beneficial to perform studies with regimens of neoadjuvant CT and SIRT to achieve better survival and minimize recurrences. 

Neoadjuvant treatment is a highly appealing option in the transplant and resection patient category, given the known poor outcome with the current standard of care (surgery alone). Upfront CRT could avoid unnecessary surgery, thereby optimizing the use of these limited and highly skilled resources, in addition to sparing a donor liver. Further, CRT may downstage a patient to a resectable or transplantable state and reduce seeding and R1 (microscopic margins) resections, theoretically allowing improved therapeutic outcomes and shorter delays due to the lack of required recovery post-surgery. Patients considering this option need to have managed expectations, as the chance of progression and dropout from the protocol is high, with only a small minority achieving transplant.

## 4. Postoperative Radiation

There exists a good amount of data, but no RCTs, to guide management in the postoperative setting (Table 3). For eCCA, the leading trials include the SWOG phase II prospective study and the John Hopkins review. SWOG S0809 was a landmark trial published in 2014 [37]. Postoperative patients were treated with GC, followed by CRT. Therefore, a multicenter single-arm trial schema and meaningful a priori success criteria were selected. Radiation obtained a 30–50% reduction in local failures, with a mOS of 35 months. Side effects were mostly related to CT and included one death from gastrointestinal hemorrhage. 

The John Hopkins trial had patients undergo, post-pancreaticoduodenectomy, 5FU and EBRT, achieving a 37-month mOS compared to an institutional cohort with only 22 months [38]. Another landmark study for eCCA was completed by Hoehn et al. [39]. A total of 8541 patients were divided into three groups: surgery only, surgery plus adjuvant CT, and surgery plus adjuvant CT and radiation. The radiation therapy group had the greatest SR among all patients, including high-risk eCCA patients. Notably, the improved survival was independent of the surgical margins, suggesting that radiation may eliminate this high recurrence factor, thereby contributing to greater LC of the disease. 

There were retrospective studies conducted for eCCA, such as Ben-David et al.’s review of cases where patients underwent adjuvant EBRT, and 54% had adjuvant CT before radiotherapy as well [40]. OS was significantly less than what was found in the previous studies discussed at 14.7 months. The largest issue was deemed to be local failure, which suggests that an adjuvant EBRT regimen may need to be more intense or radio-sensitizing agents need to be added for adequate results. A large, 1478-patient retrospective study using the National Cancer Database for eCCA was published by Shridhar et al. The addition of adjuvant radiotherapy appears to provide a significant benefit over CT alone [41]. 

Cameron et al. reviewed a cohort of 96 iCCA patients who underwent curative surgery, non-curative surgery, and palliative stenting only [42]. A total of 66% received EBRT in this retrospective review. A clinically informative finding was that the 5-year SR was 16% versus 0% for EBRT with resection versus resection alone, respectively. For patients treated with a stent, the 2-year SR was 10% versus 0% for radiation versus without radiation. 

Mukai et al. completed a study to confirm the safety and efficacy of postoperative radiation therapy, being the first to analyze a tolerance dose after surgery. At the 2-year follow-up, 72.4% of patients had survived and 47.7% were disease-free [43]. Notably, at the most recent follow-up, 16 (50%) of the patients were still alive: 8 were disease-free, 3 had local recurrences and metastases, and 2 had only local recurrences. These findings are indicative of good LC with postoperative radiotherapy, aiding in the prevention of metastasis by reducing local recurrence. 

Horgan et al. performed a 20-study meta-analysis that included 6712 patients. This team was able to assess the impact of adjuvant treatment on node-positive cases of CCA and gallbladder tumors and margin-positive subgroups. There was a non-significant effect of adjuvant treatment compared to surgery alone on OS. OS was statistically improved with either CT or CRT compared to radiotherapy alone. The patients receiving the greatest benefit were lymph node (LN)-positive or had R1 disease [44]. 

A large epidemiological multicenter review of iCCA was undertaken by Shinohara et al. using the Surveillance Epidemiology and End Results (SEER) database [27]. They demonstrated a significant advantage of surgery, radiation, and surgery plus radiation. Median survival was 3 months (no treatment), 7 months (radiation alone), 6 months (surgery alone), and 11 months (combined surgery and radiation). This trial has the usual epidemiological study caveats, including retrospective data collection, heterogeneous treatment, and, in this review, a lack of detailed CT data. It provided important confirmation that a vast majority of patients suffered poor prognosis, particularly with single-modality treatment. The best results were patients able to undergo surgery and radiation with a doubling of median survival and a hazard ratio (HR) of 0.67. However, even these patients succumbed to the disease at a median of one year after diagnosis. 

Adjuvant radiotherapy can be beneficial for minimizing local failure, contributing to decreasing the risk of metastatic disease and increasing survival time. EBRT has the potential to benefit patients with both eCCA and iCCA, although there needs to be further inquiry into dose and fractionation for optimal survival (34, 38–41). There is a lack of studies involving other RT modalities, except for pooled analysis, which does not show explicit differences between modalities.

## 5. Combined Immunotherapy and Radiotherapy

Immunotherapy is a novel area of research and is bursting with potential for various cancers, including CCA (Table 4). While only two research papers combining immunotherapy and radiotherapy in patients with CCA have been published, they both show great promise. Theories have been suggested about how radiation modifies the tumor microenvironment and sensitizes tumors to immunotherapy, possibly allowing the generation of a tumor-specific immune response [45,46].

Liu et al. published a report on three patients treated with SBRT and PD-1 blockage [45]. This report is the first of its kind involving patients who are late-stage or recurrent, all with a low tumor mutation burden, microsatellite-stable tumors, proficient mismatch repair, and negative PD-L1 expression. One patient remained progression-free by the end of the observation period, the second was progression-free for 7 months (still alive), and the last had a CR and has not had a recurrence. This is extremely encouraging for such a dismal disease and proves that the use of RT and immunotherapy should be studied further. Zhao et al. published a case series of four specific cases, which had great results, including one of the four becoming operable. Each patient underwent SBRT with varying regimens, either concurrently or with an adjuvant anti-PD-1 antibody drug, Nivolumab [46]. 

While there are many effects that radiotherapy and immunotherapy have on the body and on cancer, some known, some theorized, there are likely both synergistic and antagonistic effects. With these case reports showing such promise, it is evident how beneficial this could be for all CCA patients, even more so for inoperable cases. While there have been no clinical trials with results published yet, there are some in progress. The lack of any data above level IV relays how much this research area needs to evolve. 

## 6. Systemic Treatment in CCA

In localized CCA, there is a role for adjuvant/neoadjuvant chemotherapy. The role of systemic treatment, besides chemotherapy, is evolving in advanced/metastatic CCA, with new indications of immunotherapy and targeted treatment. 

### 6.1. Neoadjuvant Chemotherapy

The role of neoadjuvant CT in CCA is not well established. It has been used in patients with locally advanced unresectable CCA, either with CRT or chemotherapy (GC) alone, with a goal to convert unresectable disease into a potentially resectable disease and thereby improve outcomes. The data for CRT are discussed above. Most of the data for chemotherapy alone are retrospective [47,48]. A recent meta-analysis to evaluate the benefit of neoadjuvant chemotherapy in patients with resectable iCCA, involving five retrospective studies (*n* = 2412 patients), reported a better 5-year OS compared to the upfront surgery group (odds ratio (OR) = 1.27, 95% CI: 1.02–1.58). However, the R0 resection rate was lower in the neoadjuvant chemotherapy group than that in the upfront surgery group (OR  =  0.49, 95% CI: 0.26–0.91), and there were no significant differences in 1-year OS, RFS, or postoperative complications/postoperative mortality between the two groups [49,50,51,52,53,54]. Treatment with neoadjuvant chemotherapy in an initially unresectable disease should be highly individualized in the absence of level I data. Meanwhile, there are active studies recruiting patients with resectable CCA to assess the benefit of neoadjuvant chemotherapy [55,56,57,58,59]. 

### 6.2. Adjuvant Chemotherapy

After resection, local recurrence is the main cause of surgical failure in eCCA, although distant relapse does occur [60]. Local relapse rates are more common in patients with nodal spread or positive surgical margins. For iCCA, distant relapse is more common [61]. The benefits of adjuvant treatment, either CRT or CT alone, are decreased relapse rates and improved survival.

There is a general consensus among guidelines that patients with positive margins or nodes should be offered systemic therapy, either CRT or chemotherapy alone. For patients with negative margins and node-negative disease, observation or systemic CT (including CRT for eCCA) is an appropriate option [62,63]. 

A meta-analysis for patients with resected biliary tract cancer (BTC) showed that the greatest benefit of adjuvant treatment is for patients with node- or margin-positive disease (R1 resection). The ORs for patients with LN-positive disease and R1 disease were 0.49 (*p* = 0.004) and 0.36 (*p* = 0.002), respectively. Also, patients who received CT or CRT derived a statistically greater benefit compared to those who received RT alone (OR, 0.39, 0.61, and 0.98, respectively; *p* = 0.02) [44]. 

BILCAP was a phase III RCT for completely resected BTC (*n* = 447) and included patients with CCA (*n* = 368). Patients were randomized to receive oral capecitabine or undergo observation. This study was negative for the primary endpoint of OS in the ITT population; however, the prespecified sensitivity and per-protocol analyses suggested improved OS with an HR of 0.71 (95% CI 0.55–0.92; *p* = 0.010) [64]. ESPAC-3 was a phase III RCT that evaluated observation vs. adjuvant treatment (5-FU or gemcitabine) in patients with periampullary malignancies (including BTC) and found a statistically non-significant OS benefit (mOS 43 versus 35 months, HR 0.86, 95% CI 0.66–1.11). The mOS was 27, 18, and 20 months with observation, 5-FU alone, and gemcitabine alone, respectively [65]. Another phase III RCT in Japan, JCOG1202, assessed adjuvant CT with S-1 compared to resection alone in resected BTC (R0 or R1 resection). After a median follow-up of 46 months, adjuvant CT with S-1 improved OS over resection alone (3-year OS 77% versus 68%, HR 0.69, 95% CI 0.51–0.94) [66].

The ACTICCA-1 trial is an ongoing multinational, phase II RCT assessing adjuvant chemotherapy with GC compared to observation after a curative-intent resection of CCA and muscle-invasive gallbladder carcinoma [67].

### 6.3. Advanced/Metastatic Cholangiocarcinoma

The therapeutic landscape of advanced/metastatic cholangiocarcinoma continues to evolve. Historically, chemotherapy was the only option; however, immunotherapy and targeted treatment have been added to the therapeutic arsenal.

Chemotherapy with the addition of immunotherapy is the treatment of choice for the initial treatment of these patients. The FDA recently approved durvalumab with GC in patients with advanced or metastatic BTC based on the TOPAZ-1 trial [68]. TOPAZ-1 was a phase III RCT with advanced/metastatic BTC patients (75% of patients had CCA). Patients received GC with or without durvalumab and subsequently received maintenance therapy with either durvalumab or placebo. The addition of durvalumab improved OS (mOS 12.8 versus 11.5 months, HR 0.8, 95% CI 0.66–0.97), PFS, and ORR across all histologies and subtypes, with no increase in toxicity [69]. KEYNOTE-966, a phase III RCT, evaluated GC and pembrolizumab in treatment-naïve locally advanced or metastatic BTC, including CCA (78%) [70]. Patients received pembrolizumab/placebo with or without GC, followed by pembrolizumab/placebo and gemcitabine maintenance. The addition of pembrolizumab improved mOS to 12.7 vs. 10.9 months (HR 0.83, 95% CI 0.72–0.95) with durable responses. This regimen is awaiting FDA approval. 

Gemcitabine-based combinations (cisplatin, oxaliplatin, capecitabine, or S1) are preferred for patients who are ineligible for immunotherapy and do not have hyperbilirubinemia [71]. In a phase II RCT in patients with advanced/metastatic BTC, GC improved OS (mOS 11.7 vs. 8.1 months, HR 0.64, 95% CI 0.52 to 0.80, *p* < 0.001) [72]. For patients with hyperbilirubinemia, fluoropyrimidine-based combinations like FOLFOX/CAPOX are preferred.

Patients who progress on the first line should be tested for mismatch repair deficiency (dMMR)/microsatellite instability (MSI) and for specific molecular alterations. Patients with iCCA have the highest probability of an actionable genetic aberration/biomarker. In the absence of a targetable alteration and mismatch-repair-proficient status, chemotherapy is reasonable for fit patients. For patients who received GC in the first line, FOLFOX/CAPOX is an option, and vice versa. 

Based on the phase II basket trial KEYNOTE-158, pembrolizumab received tumor-agnostic approval for patients with advanced cancer with dMMR/MSI-H or TMB high (≥10 mutations/Mb) status [73]. In such patients with CCA who have not received immunotherapy in the first line, pembrolizumab is an appropriate treatment option.

The frequency of FGFR-activating mutations and gene fusions is up to 4% and 10–15% of iCCA, respectively. Mutations and gene fusions are mutually exclusive and are restricted to the small-duct subtype of iCCA [74,75,76,77]. In FIGHT-202, an open-label single-arm trial, pemigatinib (inhibitor of FGFR 1–3) was tested in previously treated advanced CCA. The ORR (primary endpoint) at a median follow-up of 17.8 months was 36% in patients with FGFR2 fusions or rearrangements. There were three CRs. The treatment was well tolerated except for specific side effects, including hyperphosphatemia (all grades, 60 percent; grade 3 or more, 12 percent), serous retinal detachment due to subretinal fluid accumulation (4 percent), and dry eye (all grades, 21 percent; grade 3 or more, 1%) [78]. Infigratinib (an FGFR1–3 selective oral TKI) has been tested in a phase II trial (*n* = 108) and showed an ORR of 23% [79]. The side-effect profile was similar to that of pemigatinib. Futibatinib (a selective, irreversible FGFR1–4 inhibitor) was tested in an open-label phase II trial, FOENIX-CCA2 (*n* = 103), in patients with iCCA with FGFR2 fusion/rearrangements with disease progression after one or more prior treatments. The ORR was 42 percent, and the treatment was well tolerated. The median duration of response was 10 months. Based on these trials, the FDA has granted accelerated approval to pemigatinib, infigratinib, and futibatinib for adult patients with previously treated, locally advanced or metastatic iCCA with FGFR2 gene fusions or other rearrangements [80,81,82].

The frequency of isocitrate dehydrogenase 1 (IDH1) mutations can vary up to 20–25% with geography and was reported at 13.1% in iCCA [83]. A phase III RCT, the ClarlDHy trial (*n* = 187), randomized patients with previously treated, advanced CCA to ivosidenib or placebo. Though the improvement in mOS in ITT was not statistically significant, when adjusted for crossover, the mOS with placebo was 5.1 months vs. 10.3 months for ivosidenib (95% CI, 3.8–7.6 months; HR, 0.49, *p*  <  .001) [84]. The FDA approved ivosidenib for patients with previously treated, locally advanced or metastatic CCA with an IDH1 mutation [85]. 

BRAFV600E mutations (up to 5%, especially in iCCA) and NTRK (less than 1%) rearrangements are other potential targets with tumor-agnostic approvals for dabrafenib plus trametinib and larotrectinib/entrectinib, respectively [86,87,88]. Targeting HER2/ERBB2 overexpression/amplification is also an area of interest. HER2 is commonly overexpressed in BTC, most commonly in gallbladder cancers and eCCA [89]. A number of studies have reported encouraging ORR data for patients with different degrees of HER2 overexpression, and this promising area continues to be examined [90,91,92,93,94].

## 7. Highlighting Current Trials and Future Trials 

Currently, there are 30 active trials in the Clinical Trials National Institutes of Health database evaluating the efficacy of radiation in CCA (headings: cholangiocarcinoma, bile duct cancer, radiation, radiotherapy; March 2024). Of these, nine are randomized, a distinct difference from the last few decades. Eight are investigating immunotherapy and radiotherapy together. There are 16 single-arm studies; while important due to controls such as no therapy being unethical, semi-known comparative arms such as chemotherapy can help to further prove or show the validity of treatments that are being investigated. A comparative arm analysis can have greater validity due to the knowledge of how typical regimens affected patients in the evaluated demographic.

The NRG G1-001 trial was based on the work by Hong et al. with doses of 37.5–67.5 Gy in 15 daily fractions for unresectable localized iCCA, which demonstrated the regimen’s safety plus a 2-year LC of 94% and a 2-year OS of 47% (18). The trial’s ambitious protocol was powered to demonstrate an OS benefit in 146 patients. Patients received six months of GC, and there was a selection criterion where any progression excluded the patient from the trial. The trial tried to answer the question of whether the most encouraging chemoradiation combination has an impact on this patient group, where ‘no surgery’ has not shown a clear benefit. Furthermore, the trial aimed to demonstrate the safety of the regimen, as there remained a high concern for toxicity, especially biliary events. The study was closed early due to poor patient accrual, once again highlighting the difficulties in conducting studies with this patient group [95]. A related trial, GI-003, investigating proton versus photon therapy (NCT03186898) is ongoing. A similar trial by a UK group was initiated, ABC-07 [96]. All patients received four cycles of GC. Staging imaging was repeated, and if there was no progression, patients were randomized to SBRT or two further cycles of GC. Radiation was either 5 fractions every second day if the tumor was small or 15 fractions daily for larger tumors. All treatments were completed within 6 months. The results of the randomized ABC-07 and SIRCCA trials are eagerly awaited.

The search for a better way to manage CCA patients has revealed important avenues of investigation; however, this disease remains difficult to manage, and the current standard of care results in some of the worst outcomes in oncology. Randomized trials are desperately needed for this niche patient group. Given a better understanding of genetics and novel immunotherapy in CCA, these new trials will hopefully lead to meaningful improvements in clinical outcomes. 

## 8. Conclusions 

Current guidelines rely on evidence-based level I data from surgery for localized disease that can be resected. However, for the majority of patients with locally advanced or metastatic disease, there has been a paucity of guidance due to the difficulty in conducting level I trials in this patient group. We reviewed a growing body of level II data that suggest that radiation and chemotherapy have a clinically important benefit. Opportunities for integration into four patient categories (unresectable or inoperable, neoadjuvant, and postoperative) are discussed. Single-modality treatment, the current standard, has the poorest outcome compared to multimodality treatment, where there is a local and overall survival advantage. The addition of immunotherapy to chemotherapy and targeted treatment has further improved outcomes.

## Figures and Tables

**Table 1:** Studies of unresectable or inoperable cholangiocarcinoma.

Author, Year	*n*	Site	Intervention	Control	Median OS (Months)	Median PFS (Months)
Phelip et al., 2014 [6]	34	CCA, GB	50Gy RT, 5-FU, Cis	GEMOX	OS: 13.5, 19.9	PFS: 5.8, 11
Tse et al., 2008 [7]	10, 31	iCCA, HCC	SBRT: 24–54 Gy, median 36 Gy, 6 fractions in 2 weeks	n.a.	IHCC: MST: 15; 1yrLC: 65%; 1yrOS: 58%.	n.r.
Frakulli et al., 2019 [8]	231	CCA	SBRT: variable	n.a.	OS: 15; 1yr: 58.3%; 2yr: 35.5%. 1yrLC: 83.4%	n.r.
Kopek et al., 2010 [9]	27	CCA	SBRT: 45 Gy in 3 fractions	n.a.	OS: 10.6	PFS: 6.7
Jung et al., 2014 [10]	58	CCA	Primary CCA; SBRT: 15–60 Gy in 1–5 fractions, median 45 Gy in 3 fractions	Same for recurrent	Overall: OS: 10; 1yr: 45%; 2yr: 20%; LC: 1yr: 85%; 2yr: 72%. Respective: OS: 5, 13.	1yrPFS: 26%; 2yrPFS: 23%.
Kozak et al., 2020 [11]	40	CCA	SBRT: 40Gy in 1–5 fractions	n.a.	OS: 23 1yrOS: 69%, 2yrOS:39%	n.r.
Baak et al., 2021 [12]	6	eCCA	After CTX (GEMCIS), SBRT: 60 Gy; 15 fractions of 3–4.5 Gy	n.a.	1yrLC: 80% 1yrOS: 100%	PFS: 14
Gkika et al., 2017 [13]	37	CCA	SBRT: median 45 Gy (25–66 Gy) in 3–12 fractions	n.a.	OS: 14 1yrOS: 56%; 1yrLC:78%	PFS: 9
Polistina et al., 2011 [14]	10	eCCA	SBRT + Gem: 30Gy in 3 fractions	n.a.	2yrOS: 80% 4yrOS: 30%	TTP: 30
Zhang et al., 2023 [15]	43	iCCA	Cyber Knife SBRT	n.a.	OS: 12; 1yrOS: 51.2%; 2yrOS: 32.6%; 3yrOS: 23.3%	PFS: 6
Chen et al., 2010 [5]	84	iCCA	EBRT: 30–60 Gy in fractions of 1.8–2.0 Gy daily; median 50 Gy	No EBRT (*n* = 49)	MST: 9.5, 5.1 1yrSR: 38.5%, 16.4%; 2yrSR: 9.6%, 4.9%	n.r.
Boothe et al., 2016 [16]	1326	eCCA	EBRT: variable	EBRT, brachytherapy	OS: 9, 11.	n.r.
Hong et al., 2016 [17]	37, 44 + 2	iCCA, HCC	PBT: max dose 67.5 Gy in 15 fractions, median 58 Gy	n.a.	IHCC: OS: 22.5; 8.4; 1yrOS: 69.7%; 2yrOS: 46.5%.	PFS: 1yr: 41.4%; 2yr: 25.7%
Hung et al., 2020 [18]	30	CCA	PBT: median 72.6 Gy; concurrent CTX (*n* = 23)	n.a.	OS: 19.3; 1yrLC: 88%; 1yrRC: 86%; 1yrDC: 68%	PFS: 10.4
Yu et al., 2021 [19]	443, 732	iCCA	EBRT: variable	SIRT: variable	MST: 13.6, 12	n.r.
Edeline et al., 2021 [20]	3990	iCCA	Pooled analysis of SIRT, TACE, HAI, EBRT	n.a.	EBRT: 18.9, SIRT: 14.1	EBRT: 15.6, SIRT:7.8
Kumar et al., 2022 [21]	16	iCCA	SIRT (min 190 Gy)	n.a.	OS: 7	n.r.
Cucchetti et al., 2017 [22]	224	iCCA	SIRT	Addition of CTX	MST: 19.5, 5.5	n.r.
Manceau et al., 2018 [23]	40	iCCA	SIRT (mean 322 Gy), CTX (25 mg/m^2^ Cis and 1000 mg/m^2^ Gem days 1 and 8, every 3 weeks; 50 mg/m^2^ Cis and 5-FU at 400 mg/m^2^ on day 1, 5-FU 2400 mg/m^2^, every 2 weeks; 1000 mg/m^2^ Gem on day 1, 100 mg/m^2^ Ox either day 1/2, every 2 weeks; Gem reduced to 300 mg/m^2^ around SIRT)	n.a.	OS: 28.6	PFS: 12.7
Edeline et al., 2020 [24]	41	iCCA	SIRT (120 Gy), Cis (25 mg/m^2^), Gem (1000 mg/m^2^, reduced to 300 mg/m^2^ just before and after SIRT, on days 1 and 8 (21-day cycle, for 8 cycles).	n.a.	OS: 22	PFS: 14
Chan et al., 2022 [25]	24	iCCA	SIRT (120 Gy) (*n* = 24), cis (25 mg/m^2^), gem (1000 mg/m^2^) on days 1 and 8 (*n* = 16).	n.a.	OS: 13.6 CTX OS: 21.6	CTX PFS: 9
Tao et al., 2016 [26]	79	iCCA	CTX, then 3D-CRT: 35–100 Gy, median 58.05 Gy in 3–30 fractions (BED 43.75–180 Gy, median 80.5 Gy)	n.a.	BED > 80.5 Gy vs <: 3yrOS: 73%, 38%; 3yrLC: 78%, 45%.	n.r.

Cholangiocarcinoma (CCA), gallbladder (GB), Gray (Gy), radiotherapy (RT), 5-fluorocil (5-FU), cisplatin (Cis), gemcitabine with oxaliplatin (GEMOX), overall survival (OS), progression-free survival (PFS), intrahepatic cholangiocarcinoma (IHCC), hepatocellular carcinoma (HCC), stereotactic body radiation therapy (SBRT), median survival time (MST), local control (LC), chemotherapy (CTX), extrahepatic cholangiocarcinoma, (EHCC), time to progression (TTP), intensity-modulated radiation therapy (IMRT), capecitabine (Cap), photodynamic therapy (PDT), disease-free survival (DFS), external beam radiation therapy (EBRT), stereotactic ablative radiation therapy (SABR), hypo-fractionated radiation therapy (HF-RT), proton beam therapy (PBT), regional control (RC), distal control (DC), Yttrium-90 microspheres (SIRT), oxaliplatin (Ox), Child–Pugh Score (CPS), 3-dimensional conformal radiation therapy (3D-CRT), biological equivalent dose (BED), gemcitabine (Gem), transarterial chemoembolization (TACE), hepatic arterial infusion (HAI).

**Table 2:** Studies of preoperative radiation and transplantation cholangiocarcinoma.

Author, Year	*n*	Site	Treatment	Comparison	Median OS (Months or %)	Median PFS (Months or %)
Heimbach et al., 2004 [28]	56	eCCA	Neoadjuvant EBRT (45 Gy in 30 fractions, 2 Gy/fraction), brachytherapy (I192-20–30 Gy, 2–3 weeks after EBRT), 5-FU (500 mg/m^2^ daily for 3 days [EBRT], 225 mg/m^2^ daily [brachytherapy]), Cap (2000 mg/m^2^/day in 2 divided doses, 2/3 weeks), then transplant	n.a.	5yrSR: 54%, 64% (48 operatively staged), 84% (34 with negative staging operations); 1yrSR transplant: 88%; 2yrSR transplant: 82%	n.r.
Azad et al., 2020 [29]	237	hCCA	Neoadjuvant EBRT (45 Gy in 30 fractions, 2 Gy/fraction), brachytherapy (I192- 20–30 Gy, 2–3 weeks after EBRT), 5-FU (500 mg/m^2^ daily for 3 days [EBRT], 225 mg/m^2^ daily [brachytherapy]), Cap (2000 mg/m^2^/day in 2 divided doses, 2/3 weeks), then transplant	De novo/PSC	1yrSR: 92%, 5yr SR: 68%, 10yr SR: 60%. De novo: 1yrSR: 91%, 5yr SR: 58%, 10yr SR: 49%. PSC: 1yrSR: 93%, 5yr SR: 74%, 10yr SR: 67%.	n.r.
Murad et al., 2012 [30]	287	eCCA	EBRT (99%) (45 Gy), brachytherapy (75%) (20 Gy), radio-sensitizing therapy (98%), and/or CTX (65%), transplant (88.5%)	n.a.	Intent-to-treat SR: 2yr: 68%; 5yr: 53%	Post-transplant, recurrence-free survival rates: 2yr: 78%; 5yr: 65%
McMasters et al., 1997 [31]	91 (40)	CCA	Chemoradiation (5-FU 300 mg/m^2^ per day), EBRT (1.8 Gy/day to a total dose of 50.4 Gy (*n* = 5) or 45 Gy (*n* = 2)), resection (9)	Resection (31)/palliative (51)	pCR: 3 chemoradiation: 100% margin-negative. No chemoradiation: 54% margin-negative.	n.r.
Loveday et al., 2018 [32]	43	eCCA	Chemoradiation (55–75 Gy in 1.5 Gy BID in 4–5 weeks with 800 mg/m^2^ Cap), staging, maintenance chemotherapy (GEMCIS 1000 mg/m^2^ and 25 mg.m^2^ days 1/8), transplantation	n.a.	OS: 16.4. 1yrSR: 70.6%. 2yrSR: 35.3%. Post-transplant: 1yrSR: 83.3% 2yrSR: 55.6%.	PFS: 11.5
Welling et al., 2014 [33]	17	eCCA	SBRT: 50–60 Gy in 3–5 fractions in 2 weeks. 1 week after capecitabine: 1330 mg/m^2^/day until transplant.	n.a.	1yrSR: 83%	n.r.
Sarwar et al., 2021 [34]	37	iCCA	SIRT: Median 155 Gy (120–200 Gy)	n.a.	OS: 22 1yrOS: 60%; 2yrOS: 40%	PFS: 5.4
Ito et al., 2022 [35]	19 (30)	hCCA, iCCA	5-FU, Cap, or Gem with oxaliplatin, leucovorin, and Cis, RT, TACE, or RFA were used for some patients. Current protocol: iCCA <6 cm or hCCA and iCCA ≥6cm are treated with SBRT of 40 Gy in 5 fractions and TACE. GEMCIS for neoadjuvant chemotherapy (NAC) until LT.	Neoadjuvant CTX, neoadjuvant LT, combination	Combination: 5yr OS: 88% (hCCA), 100%(iCCA). Not combination: 5yr OS: 9% (hCCA), 41% (iCCA).	n.r.
Hong et al., 2011 [36]	20, (37)	hCCA, iCCA	CTX or CRT was given before and/or after surgery. 5-FU or Cap with oxaliplatin, leucovorin calcium, and gemcitabine hydrochloride was used for adjuvant and neoadjuvant protocols.	Transplant (38), partial hepatectomy (19)	Transplant: 47% (neoadjuvant and adjuvant), 20% (no therapy), 33% (adjuvant therapy). 5yr OS: 34% (iCCA), 29% (hCCA).	5yr recurrence-free survival: 33% (transplant), 0% (partial hepatectomy).

Cholangiocarcinoma (CCA), Gray (Gy), year (yr), radiotherapy (RT), 5-fluorouracil (5-FU), cisplatin (Cis), overall survival (OS), progression-free survival (PFS), intrahepatic cholangiocarcinoma (iCCA), stereotactic body radiation therapy (SBRT), median survival time (MST), survival rate (SR), local control (LC), chemotherapy (CTX), extrahepatic cholangiocarcinoma, (eCCA), capecitabine (Cap), external beam radiation therapy (EBRT), pathological complete response (pCR), Yttrium-90 microspheres (SIRT), biological equivalent dose (BED), gemcitabine (Gem), twice per day (BID), primary sclerosing cholangitis (PSC), gemcitabine and cisplatin (GEMCIS), transarterial chemoembolization (TACE), radiofrequency ablation (RFA), radiation therapy (RT), chemoradiotherapy (CRT).

**Table 3:** Studies of postoperative radiation cholangiocarcinoma.

Author, Year	*n*	Site	Treatment	Comparison	Median OS (Months or %)	Median PFS (Months or %)
Ben-Josef et al., 2015 [37]	79	CCA, GB	After resection, 4 cycles of Gem (1000 mg/m^2^ days 1, 8), Cap (1500 mg/m^2^ days 1–14) every 21 days, then concurrent Cap (1330 mg/m^2^/day) and RT (45 Gy to lymphatics; 54–59.4 Gy to tumor bed)	n.a.	2 yr SR: 65%; 67% and 60% in R0 and R1. OS: 35 (R0, 34; R1, 35). Local, distant, and combined relapse in 14, 24, and 9 patients.	n.r.
Hughes et al., 2007 [38]	34	CCA	PD, chemoradiation (median dose was 50.4 Gy (40–54 Gy)). Concurrent 5-FU, followed by maintenance chemotherapy	n.a.	OS: 36.9. 5 yr SR: 35%; 5yrSR: 100% (LN-), 24% (LN+); 5 yr LC: 70%. SR: 36.9 (PD alone), 22 (PD+adjuvant therapy).	n.r.
Hoehn et al., 2015 [39]	8741	CCA	Surgery, adjuvant chemotherapy; surgery alone	Surgery, adjuvant chemoradiation	MST: 24.84, 33.6, 33.12	n.r.
Ben-David et al., 2006 [40]	81	eCCA	Adjuvant EBRT: median 58.4 Gy (23–88.2 Gy) (2Gy/fraction), 54% concurrent chemotherapy	n.a.	OS: 14.7	PFS: 11
Shridhar et al., 2022 [41]	1478	eCCA	Various RT after adjuvant chemotherapy: 25–33 fractions, 45–59.4 Gy	Adjuvant chemotherapy	RT: OS: 34, 5yrOS: 33%. No RT: OS: 27, 5yrOS: 24%.	n.r.
Cameron et al., 1990 [42]	96	CCA	Surgery, RT (66%)	Stenting, RT (66%)	1, 3, 5, 10yr SR (entire group) 49%, 12%, 5%, 2%. 1, 3, 5, 10yr SR (resected group): 66%, 21%, 8%, 4%. 1, 3, 5yr SR (stented group): 27%, 6%, 0%.	n.r.
Mukai et al., 2019 [43]	32	CCA	Surgery, adjuvant RT (median dose 50 Gy)	n.a.	2yr OS: 72.4%. LC: 65.3%; MST: 40.	DFS: 47.7%
Horgan et al., 2012 [44]	6712	BTC	Adjuvant chemotherapy or adjuvant RT (systematic review + meta-analysis)	Adjuvant chemoradiation	Non-significant improvement in OS with any therapy compared with surgery alone. CTX/CTX+RT statistically better than RT. Adjuvant therapy best in LN+ and R1 disease.	n.r.
Shinohara et al., 2008 [27]	3839	iCCA	Surgery alone (25%), RT alone (10%), surgery + adjuvant RT (7%)	none (58%)	OS: 6, 7, 11, 3	n.r.

Cholangiocarcinoma (CCA), gallbladder (GB), pancreaticoduodenectomy (PD), Gray (Gy), radiotherapy (RT), 5-fluorouracil (5-FU), lymph node (LN), overall survival (OS), progression-free survival (PFS), intrahepatic cholangiocarcinoma (iCCA), biliary tract cancer (BTC), median survival time (MST), local control (LC), chemotherapy (CTX), extrahepatic cholangiocarcinoma, (eCCA), capecitabine (Cap), external beam radiation therapy (EBRT), gemcitabine (Gem), not reached (n.r.).

**Table 4:** Radiation and immunotherapy in CCA.

Author, Year	*n*	Site	Treatment	Median OS (Months)	Median PFS (Months)
Liu et al., 2019 [45]	3	CCA	SBRT (Cyber Knife 52 Gy/4 fractions or 55 Gy/5 fractions), PD-1 blockade (nivolumab 200 mg every 2 weeks, 15 cycles)	Patient C: CR: 11	Pt A: PFS: 13 Pt B: PFS: 7, OS: 13
Zhao et al., 2021 [46]	4	CCA	SBRT (25–60 Gy in 5–12 fractions), anti-PD-1 antibody (Nivolumab 140–200 mg)	All cases controlled, 1 became resectable; OS: 12+	n.r.

Cholangiocarcinoma (CCA), overall survival (OS), progression-free survival (PFS), stereotactic body radiation therapy (SBRT), patient (Pt), not reached (n.r.).
